# Culturing the Uncultured: Risk versus Reward

**DOI:** 10.1128/mSystems.00130-19

**Published:** 2019-05-21

**Authors:** J. Cameron Thrash

**Affiliations:** a Department of Biological Sciences, University of Southern California, Los Angeles, California, USA

**Keywords:** cost-benefit analysis, cultivation, environmental microbiology

## Abstract

Isolation of new microorganisms is challenging, but cultures are invaluable resources for experimental validation of phenotype, ecology, and evolutionary processes. Although the number of new isolates continues to grow, the majority of cultivars still come from a limited number of phylogenetic groups and environments, necessitating investment in new cultivation efforts. While most microbiologists probably agree that axenic cultures have great value, we need to collectively put our money where our mouth is. I propose that we examine cultivation from the perspective of expected value to rationally incorporate risks and rewards of isolating new microbes. If we can even broadly constrain the cultivation probability and relative values of isolates, we can use this information to evaluate and improve experimental design. There are numerous scenarios for which isolation projects have positive expectations and therefore represent a sound investment.

## Abstract (precis)

Isolation of new microorganisms is challenging, but cultures are invaluable resources for experimental validation of phenotype, ecology, and evolutionary processes. Although the number of new isolates continues to grow, the majority of cultivars still come from a limited number of phylogenetic groups and environments, necessitating investment in new cultivation efforts.

## PERSPECTIVE

Bringing new microorganisms into culture can be difficult. It relies upon creative, well-researched, and often lucky (shall we say educated?) guesses about the types of conditions that a particular microbe needs for growth. New cultivation efforts also require persistence because most attempts to isolate organisms not yet in culture fail. Yet the value of having a living organism to query experimentally provides unmatched opportunities to understand the microorganism in question and can lead to very important ancillary applications.

As someone whose Ph.D. and postdoctoral training included classical microbial physiology and isolation of novel taxa, microbe hunting has been integral to my lab’s research plan from day 1. Since 2013, we have developed and refined our modified high-throughput cultivation protocol and medium recipes based on many successful models (1–7) and careful examination of local seawater chemistry. Our first suite of artificial media led to the cultivation of many highly abundant taxa from the coastal Gulf of Mexico, a region with few cultivated representatives from the most frequently occurring clades (8). These include SAR11, SAR116, and HIMB11-type *Roseobacter* organisms in the *Alphaproteobacteria*; a host of taxa from the oligotrophic marine *Gammaproteobacteria* groups, like OM60 (4); and (since we sampled in regions with large salinity fluctuations) cosmopolitan “freshwater” bacterioplankton like *Limnohabitans*, BAL58, and the MWH-UniP4 *Betaproteobacteria* groups. For many abundant but traditionally hard-to-cultivate taxa, like SAR11, this was also the first demonstration that an artificial medium (instead of sterilized natural seawater [see, e.g., reference 2]) could effectively work for isolating new strains (8). Our most important success story to date has been isolating the first representative of the so-called freshwater SAR11, or LD12 clade, some of the most abundant freshwater bacteria globally, which also inhabit brackish environments (9). Cultivation and subsequent genome sequencing of that representative, “*Candidatus* Fonsibacter ubiquis” LSUCC0530, yielded important new data about the range of salinities in which this organism can grow and provided a concise, testable hypothesis concerning the evolutionary divergence of the LD12 clade from its marine SAR11 cousins (9).

**FIG 1:**
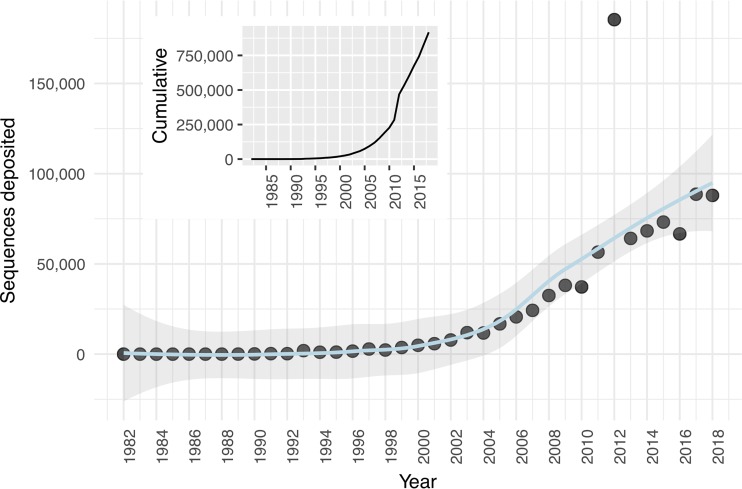
Isolate 16S rRNA gene sequences deposited into GenBank by year. Nonlinear regression is shown in blue, with 95% confidence intervals shaded. (Inset) Cumulative isolate 16S rRNA gene sequences in GenBank over time. The code for Entrez searches and figure generation is available at https://github.com/jcthrash/16S_deposition.

We are not alone in enthusiastically bringing new microbes into culture. Isolation work in many labs continues at a considerable pace. If we take deposition of cultivar 16S rRNA gene sequences into GenBank as a proxy, isolation efforts have doubled over the last decade, and in both 2017 and 2018, roughly 88,000 sequences were deposited (Fig. 1). But these data belie a strong anthropocentric cultivation bias. The majority of cultivars represent taxa from only a few habitats (like humans [10, 11]), and in general, most microorganisms remain uncultivated (11). Environments like soil, the terrestrial subsurface, marine sediments, and many aquatic systems ought to receive vastly more cultivation attention (11).

What forces restrain more fervent hunting for new microbes, especially in underexplored environments? Training and infrastructure requirements for protocols like ours, which include specialized equipment, molecular methods, and stringent contamination controls, can represent barriers to entry. We have presented alternative methodology that circumvents some of these barriers (12) and have even been successful at modifying our cultivation protocol for deployment in the context of undergraduate teaching labs (13). Perhaps the most pernicious barrier to more widespread microbial isolation efforts is the difficulty in obtaining funding to support this kind of resource development work. If we want cultures from currently uncultivated groups, we need to fund people to conduct isolation experiments from environments other than the human gut (11). However, I know that many of us have frustrating stories of proposal reviewers lamenting the futility of new isolation attempts. Although one might view the large numbers of sequence depositions (Fig. 1) and the vast number of still-uncultivated groups (11) as indication that isolation work does not yield novel taxa, considerable evidence argues against such a lugubrious conclusion. Researchers continually bring the uncultured into culture (1, 3, 8, 9, 14–22), and we need more liberal application of these methods to undersampled environments.

One might concede that isolation work succeeds in bringing new microorganisms into culture but continue to argue against funding cultivation based on it being too risky. However, there are two major problems with this argument: first, it implies that we actually know the risks; second, it ignores the value of the reward. My lab has begun trying to quantify cultivation risk (or probability) by comparing how frequently we cultivate specific microorganisms of a given group (to the genus and/or species levels) with their abundances in nature. So far, we see a wide range of outcomes. For example, across 17 experiments with 5 96-well plates each, we isolated HIMB11-type *Roseobacter* spp. (*Alphaproteobacteria*) 24 times out of the estimated 84 times that they would have been in our culture wells, giving that group a cultivation probability (c*P*) of 28.2%. We isolated SAR11 subclade IIIa less frequently, despite its greater abundance: 6 isolates out of the estimated 410 possible, or a c*P* of 1.46%. We also have groups with c*P* values of >60%, as those who use selective media or conduct enrichment-based isolation will not find surprising.

The absence of a relationship between relative abundance and cultivation probability across taxa suggests that dilution-based cultivation is nonrandom, but what factors govern these varied cultivation probabilities? Certainly, cultivation success can hinge on how well media mimic the natural environment of the organism (21). Yet, even when we have a successful growth medium, some isolates appear to have inherently lower cultivation probabilities than others. Biological explanations for these differences include variable dormancy rates and stochastic release from these states (15) as well as subpopulation phenotypic specialization (23) that we need to (and with cultures can) test.

Regardless of the mechanism underlying variations in c*P*’s, we can at least constrain c*P*, and conversely, the risk, for many microorganisms already in culture. However, risk means little if we ignore the value of the outcome. A way to quantify risk versus reward is through the perspective of expected value (EV, or “expectation”):

EV=(i⋅cP)+−e(1−cP)

In this case, *i* is the value of an isolate and *e* is the cost of the experiment or project (thus expressed as a negative value). To determine whether the project is too risky, we need to calculate whether the numbers lead to a positive or negative expectation. If the expectation is positive, then the process over time yields more value than it costs, making it a good bet. We have discussed estimating c*P* above, which leaves the question of how to quantify the value (*i*) of an isolate. Since we are talking about cultivation risk in terms of monetary expenditures at the funding table, we should find a way to estimate the monetary value of isolates as well. We can use a number of metrics, such as downstream grant dollars obtained, commercialization potential, market value of small molecules produced, etc. How much do you think the first *Lokiarchaeota* isolate is worth? How much was the first Streptomyces griseus, Escherichia coli, Thermus aquaticus, or Shewanella oneidensis worth with the metrics above? It may appear a fruitless attempt to place a price on the knowledge and research value gained from these microorganisms. However, we need to set only minimum bounds for the value of future isolates to use expectation calculations effectively. If we ignore commercial opportunities and consider research investment value only in dollars, it is extremely plausible that a new isolate leads to a couple of grants worth $500,000 each, to say nothing of the much larger scientific value of the knowledge obtained by studying the organism. Appraisal using grant dollars alone therefore likely represents a minimum value for the organism, but it provides us a number with which to evaluate expectation.

If we can constrain *i* and c*P* for a given microbe, the framework of expectation makes it much easier to determine what we should invest in its isolation. Using the strain-specific c*P* discussed above, let us imagine an entire experiment devoted to culturing a SAR11 IIIa strain that we valued at $500,000. Now, because of its low c*P* (1.46%), we should not spend more than about $7,400 on this experiment to maintain a positive expectation. However, we can add expectations from multiple different isolates from the same project. This can have important effects on the bottom line, both because a high positive expectation in one category can overcome a low expectation in another and because we can amortize the cost over multiple isolates, *x*, as follows:

$${\text{EV}}_{1}=(i_{1}\cdot \text{c}P_{1})+[-e/x\cdot (1-\text{c}P_{1})]$$

$${\text{EV}}_{2}=(i_{2}\cdot \text{c}P_{2})+[-e/x\cdot (1-\text{c}P_{2})]$$

$${\text{EV}}_{\text{proj}}{}_{\text{ect}}=\sum_{x=1}^{n}{\text{EV}}_{x}$$

If the same experiment aimed at isolating a SAR11 IIIa strain additionally brings in a HIMB11 strain, which has a higher c*P*, even though it may be less valuable (let us use an *i* of $50,000), the expectation stays positive with a project cost of $25,000, compared to the $7,400 if we consider SAR11 IIIa alone.

We can also use expectation in the reverse to evaluate whether our experimental design is feasible based on an organism’s c*P*. Let us say that I want to isolate a SAR11 IIIa strain, present at 20% community abundance. If I inoculate 500 wells with a dilution of my natural community to 1 cell per well, I should expect ∼100 wells with IIIa cells. Because the c*P* is 1.46%, I should expect to isolate only one IIIa cell from that experiment. There are numerous operational reasons why we might not want to cut it that close. To hedge our bets, we should probably run 5 to 10 of these experiments. Can we do that for the $7,400 (including person hours, reagents, etc.), which would keep the expectation positive? The power of added EV from other isolates comes into play here too, because maybe this set of experiments is not feasible at $7,400, but it might be at $25,000 if we consider that the same experiment will bring in multiple taxa.

The more isolates obtained by the project, the more we amortize the risk through decreasing costs per isolate. High-throughput cultivation experiments isolate hundreds or thousands of taxa at a time, drastically reducing per-isolate costs. Our recent 17-experiment project was funded by an ∼$130,000 grant and yielded 328 isolates, meaning that each isolate (including labor, overhead, propagation, cryopreservation, DNA extraction, PCR, identification, and comparison with amplicon data) cost approximately $400. I am confident that automation and other upgrades, like direct PCR, will bring this number down substantially as well.

To evaluate whether any given real-world cultivation project makes sense from an investment perspective, we need to estimate the overall expectation in the context of hundreds or thousands of taxa with differing values of *i* and c*P*, while distributing the cost across the total number of isolates. A critical component will be the negative effect of low-value taxa, which ought to vastly outnumber the high-value taxa, on the overall project expectation from many environments. However, in some places, like deep ocean sediment, where the bulk of the community is uncultivated (11), low-value taxa may actually represent a minor fraction of the haul.

**FIG 2:**
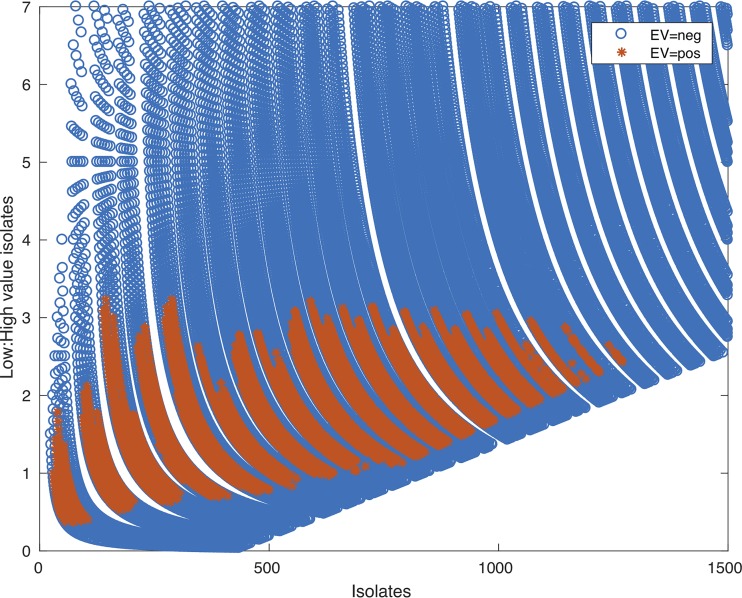
Possible EV outcomes for a hypothetical cultivation project with nine (*i* · c*P*) categories. The ratio of low-value to high-value isolates is plotted against the total number of isolates generated by any given combination of isolates in each category. Positive (pos) or negative (neg) expectation is orange or blue, respectively. Code for this simulation, including all isolate categories and number combinations, is available at https://github.com/jcthrash/ev_sim_100K.

I simulated the effects of various isolation outcomes on project expectation using nine hypothetical categories of isolates spanning different *i*’s and c*P*’s. The highest-value category ($10 million) also had the lowest c*P* (0.1%), which could mimic something like a *Lokiarchaeota* isolate. Holding the nine (*i* · c*P*) categories and *e* constant ($100,000), I varied the numbers of isolates in each category and calculated the overall project EV over thousands of different category combinations (Fig. 2). From this simulation, three things become apparent: (i) the distribution of isolates in different (*i* · c*P*) categories greatly affects the project expectation, (ii) high ratios of low-value isolates negatively impact expectation, and (iii) positive expectations are possible across a wide range of (*i* · c*P*) combinations and total numbers of isolates. This means that there are myriad scenarios where cultivation is worth it, and therefore, we cannot make an *a priori* blanket statement that any given isolation project is too risky.

Modeling expectation across a variety of cultivation scenarios certainly needs more work than what I have put forth here. An important challenge lies in estimating the c*P*’s of uncultivated taxa, and each environment will need input from experts on the microbiology of that system and the organisms in question to estimate these probabilities most effectively. A systematic examination of cultivation studies that yielded first-of-their-kind isolates, combined with information about the abundances of uncultivated taxa in the starting samples (20, 22), might also help us generate upper and lower c*P* bounds. Of particular relevance are studies that successfully utilized meta-omic data to construct appropriate cultivation conditions for isolating new taxa (e.g., see references [Bibr B24][Bibr B25][Bibr B26]). The success of such cases means that some uncultivated taxa may have quite high c*P*'s and therefore minimal risk. Additionally, we need to decide how we evaluate the value of isolates—essentially the returns on scientific investment in culturing. Axenic cultures can generate immense returns, because they provide knowledge about the natural world and can be shared and propagated across many different laboratories and even classrooms. I believe that a community dialog about funding new isolation attempts would benefit from rationally estimating risk versus reward. Given the vast number of uncultured organisms from so many environments that we can pursue domesticating, perhaps the framework of expectation can help facilitate community buy-in for what my colleague Paul Carini calls a “cultural renaissance.”
